# Iterative genetic testing identifies *SAMHD1* deficiency caused by a homozygous balanced translocation

**DOI:** 10.70962/jhi.20260044

**Published:** 2026-08-26

**Authors:** Paul J. Baker, Yaoyuan Zhang, Imogen Bishop, Madeline L. Cleveland, Alexandra L. McAllan, Georgina E. Hollway, Ravikiran Vedururu, Amit Kumar, Raj Krishnaswamy, Ken L. Wan, Peter A. Kaub, Emma L. Brown, Dhanya Lakshmi Narayanan, Ira W. Deveson, CIRCA, AADRY, Peter Gowdie, William D. Renton, Samar Ojaimi, Andrew P. Fennell, Seth L. Masters

**Affiliations:** 1 Centre for Innate Immunity and Infectious Diseases, Hudson Institute of Medical Research, Clayton, Australia; 2 Department of Molecular and Translational Science, Faculty of Medicine, Nursing and Health Sciences, Monash University , Clayton, Australia; 3 Immunology Division, The Walter and Eliza Hall Institute of Medical Research, Parkville, Australia; 4 Department of Medical Biology, University of Melbourne, Parkville, Australia; 5 Garvan Institute of Medical Research, Darlinghurst, Australia; 6 School of Clinical Medicine, Faculty of Medicine and Health, UNSW , Sydney, Australia; 7 Department of Diagnostic Genomics, Monash Health Pathology, Monash Medical Centre, Clayton, Australia; 8 School of Medicine, College of Health, Adelaide University, Adelaide, Australia; 9 Monash Genetics, Monash Health, Clayton, Australia; 10 School of Clinical Sciences at Monash Health, Faculty of Medicine, Nursing and Health Sciences, Monash University, Clayton, Australia; 11 The Clinical Immunogenomics Research Consortium Australasia, Darlinghurst, Australia; 12 Australian Autoinflammatory Diseases Registry, Clayton, Australia; 13 Department of Paediatric Rheumatology, Monash Children’s Hospital, Clayton, Australia; 14 Rheumatology Service, Department of General Medicine, The Royal Children’s Hospital, Parkville, Australia; 15 Department of Diagnostic Immunology, Monash Health Pathology, Monash Medical Centre, Clayton, Australia

## Abstract

We present a patient with complex symptoms, including those consistent with familial chilblain lupus (FCL). A de novo likely pathogenic variant in *MN1* explains observed microcephaly, sensorineural hearing loss, growth restriction, and mild dysmorphism, but not perniosis with acral autoamputation, small joint arthritis, and immune abnormalities. Transcriptomics revealed a type I IFN signature and strong downregulation of *SAMHD1*, which is associated with a spectrum of interferonopathies, including FCL. RNA reads from only the first 4 exons of *SAMHD1* were detectable, with no coverage of exon 5 onward. Subsequent long-read genome sequencing identified a homozygous, balanced, reciprocal translocation from the *SAMHD1* locus on chromosome 20 (q11.23) to chromosome 17 (p11.2). Utilizing an iterative genetic testing approach encompassing exomic, transcriptional, and genomic readouts was invaluable for elucidating the uncommon structural variation carried by this patient. Autozygous balanced, reciprocal translocations are extremely rare, and this appears to be the first case of any inborn error of immunity attributed to this mode of inheritance.

## Teaser

Baker et al. report an inborn error of immunity resulting from a genomic translocation. A 13-year-old male is identified with complex symptoms including those consistent with familial chilblain lupus. Iterative genetic testing identifies loss of expression of the interferonopathy gene *SAMHD1* resulting from a unique homozygous balanced translocation between chromosome 17 and chromosome 20.

## Introduction

Excessive and sustained production of the antiviral type-I IFN (IFN-I) family of cytokines can lead to inflammation and cell death, which, if left unchecked, may result in an autoinflammatory feedback loop and neurological abnormalities. In the absence of an infectious or traumatic trigger, these IFN-I–driven disorders are categorized as interferonopathies, a subset of autoinflammatory diseases. Familial chilblain lupus (FCL) is a rare interferonopathy that typically presents from early life, in contrast to spontaneous chilblain lupus, which predominantly manifests in middle-aged women. FCL may present with cutaneous violaceous patches and papules or plaques on the nose, ears, hands, and feet, which are exacerbated by exposure to cold temperatures. These erythematous lesions can progress to nail loss and tissue disfigurement in the affected areas (1). Autosomal dominant inheritance of FCL has been observed in patients carrying heterozygous variants in *STING1* (2) and *TREX1* (3, 4, 5, 6, 7, 8, 9, 10, 11, 12, 13, 14). *SAMHD1* has previously been associated with autosomal dominant FCL (15, 16); however, population data are no longer supportive of the association described by Ravenscroft and colleagues, while the case described by Linggonegoro and colleagues lacks supporting genetic data. Monoallelic or biallelic variants in *TREX1*, biallelic variants in *SAMHD1*, or variants in a number of other genes have also been identified as monogenic causes of Aicardi–Goutières syndrome (AGS) (17). More complex AGS patients may additionally present with symmetric intracranial calcification, systemic inflammation, arthritis, and complex neurological symptoms.

*SAMHD1* encodes the sterile alpha motif (SAM) domain and histidine-aspartic acid (HD) domain-containing protein 1 (SAMHD1), which possesses deoxynucleotide triphosphate (dNTP) triphosphatase activity. SAMHD1 limits the cellular pool of free dNTPs, thereby restricting available material for unregulated activation of nucleic acid–sensing pattern recognition receptors (18, 19). This has been reported across many SAMHD1-deficient individuals to result in systemic overproduction of IFN-I and elevated and chronic transcription of IFN-stimulated genes (ISGs) (20, 21, 22). Recently, Rabinowitz and colleagues have shown in SAMHD1-deficient THP-1 monocytes that this elevated ISG transcription is dependent on functional detection of double-stranded DNA (dsDNA) by cyclic GMP-AMP synthase and stimulator of IFN genes (STING). Correspondingly, knocking out the cytoplasmic RNA sensors melanoma differentiation-associated protein 5 (MDA5) and retinoic acid–inducible gene I (RIG-I) had no effect on ISG levels in the THP-1 cells, suggesting that, at least in monocytes, the lack of SAMHD1 activity is primarily detected as cytoplasmic accumulation of dsDNA (19).

We present here the unique case of a translocation event between chromosomes 17 and 20 resulting in disruption of *SAMHD1*. The proband presents with symptoms consistent with FCL, but without the neurological complications typical of AGS. The patient also carries a de novo likely pathogenic variant in *MN1*, adding further complexity to the clinical presentation.

## Results

**Figure 1.**
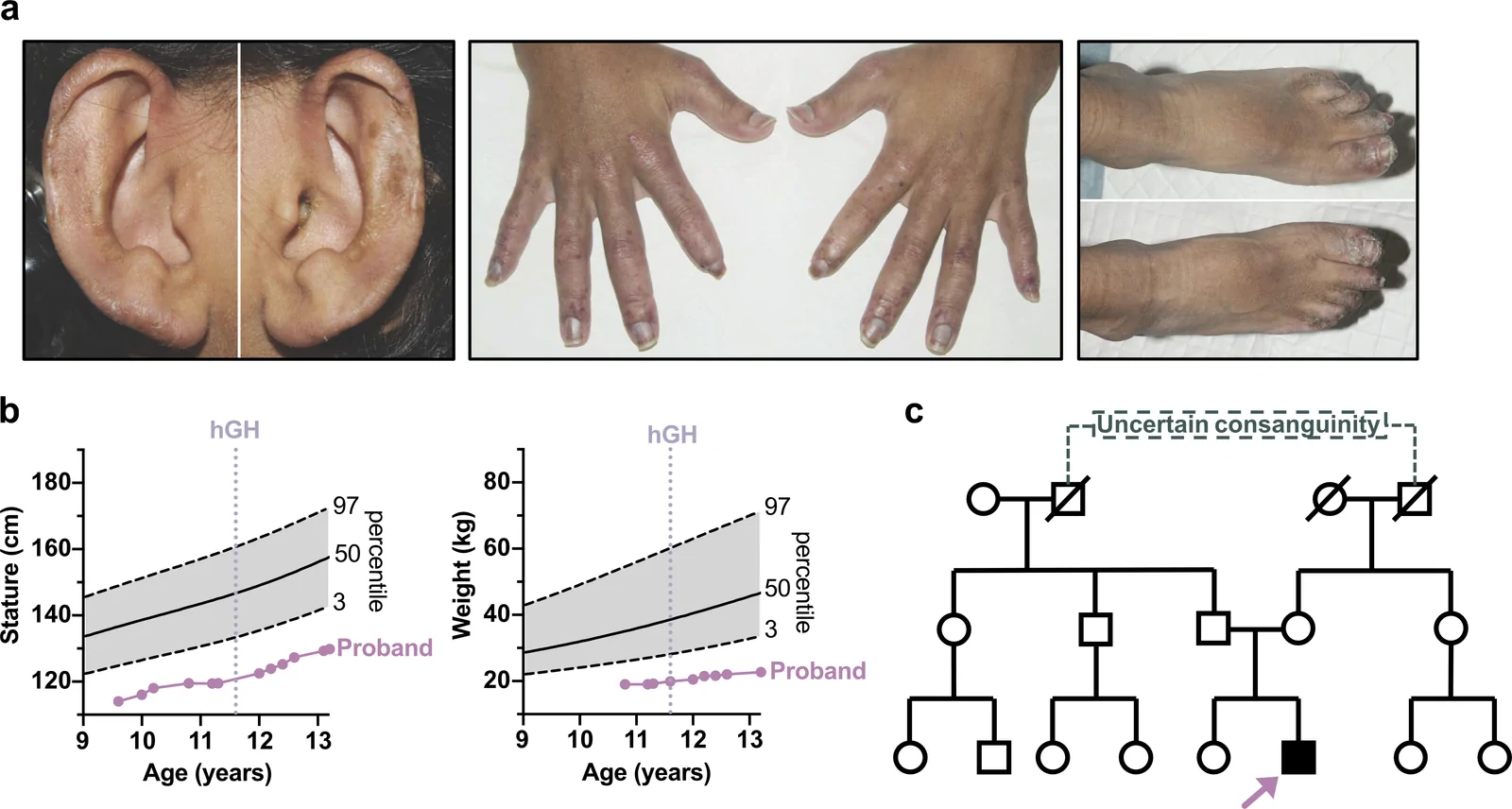
**The proband presented with symptoms consistent with FCL. (a)** Photographs of the proband depicting violaceous papules and erosion of acral tissues prior to treatment. Erosion and scarring of both ears, perniosis and tapering of the fingers, and perniosis and erosion of the toes are shown. **(b)** Growth charts tracking stature (top) or weight (bottom) of the proband (pink line) from 9.6 to 13.2 years old. Commencement of recombinant human growth hormone (hGH) (violet line) is indicated. Percentiles from the Centre for Disease Control are depicted by the grey shaded area. **(c)** Pedigree of the patient presenting FCL-like disease (indicated by pink arrow) and potential fourth-degree consanguinity as reported by the family. Filled and open symbols represent an FCL-like or normal phenotype, respectively.

A 13-year-old male presented with early-onset severe perniosis with autoamputation of several toes, mild erosion of other extremities including fingers and ears, and widespread small joint arthritis (active joint count 14). He had been affected by severe perniosis from late infancy (Fig. 1 a). He had mild facial dysmorphism, microcephaly (Z-score −4), severe sensorineural hearing loss and unilateral myopia in addition to growth restriction (height Z-score −3.2) despite treatment with recombinant human growth hormone (Fig. 1 b). Magnetic resonance imaging of the brain showed no signs of cerebral calcifications or other lesions, and the patient had no other remarkable neurological symptoms and an age-appropriate intellect. High-resolution computerized tomography scanning revealed no signs of interstitial lung disease. Clinical records reported late eruption of permanent teeth, laryngeal inflammation, mild mitral regurgitation, and resolved eczema, as well as immune abnormalities characterized by accelerated erythrocyte sedimentation rate (ESR), but normal C-reactive protein, persistent mild anemia but normal ferritin levels, mild neutropenia, natural killer (NK) cell lymphopenia, hypergammaglobulinemia, and elevated CD19^+^ B cells, although his switched memory B cell counts (CD19^+^, CD27^+^, IgM^−^, and IgD^−^) were very low (Table 1). Despite the observed hypergammaglobulinemia, autoantibody titers for antinuclear antibodies, anti-extractable nuclear antibodies, anti-dsDNA, anti-proteinase-3, anti-myeloperoxidase, and antiphospholipid antibodies were normal. Both parents were phenotypically normal at consultation and reported an uncertain degree of consanguinity through their respective paternal lines (Fig. 1 c). Microarray analysis of the proband revealed a male molecular karyotype with no clinically significant copy number variations. However, extensive long continuous stretches of homozygosity were detected, representing ∼6.6% of the genome, indicative of such consanguinity.

**Table 1.** Hematological and immunological findings of the proband over time

Parameter (units)	Age (years)	13.96	14.08	14.2	14.45	14.78	14.98	15.16	15.54
	Normal range	Pre-tofacitinib			Post-tofacitinib				
Hemoglobin (g/L)	130–180	**108**	**109**	**112**	**114**	**115**	130	**124**	**119**
Ferritin (µg/L)	20–340	63	N/A	N/A	N/A	25	N/A	24	N/A
Mean corpuscular volume (fl)	78–98	80	81	81	82	83	82	80	81
Total leukocytes (×10^9^/L)	4–11	5.8	5.7	5.5	5.5	6	4.1	4.1	5.5
Total lymphocytes (×10^9^/L)	1–4	2.5	2.8	3	3	3.5	2.9	2.3	2.6
CD19^+^ B cells (×10^6^/L)	110–570	*640*	*836*	*1,029*	*1,257*	*1,319*	N/A	N/A	*895*
Switched memory B cells* (10^6^/L)	30–110	N/A	N/A	**7**	N/A	N/A	N/A	N/A	**25**
NK cells** (×10^6^/L)	70–480	**27**	**34**	**41**	**44**	125	N/A	N/A	88
Neutrophils (×10^9^/L)	2–8	2.8	2.4	2	**1.9**	2	**0.94**	**1.4**	2.3
Platelets (×10^9^/L)	150–450	*506*	*502*	*496*	*461*	*514*	437	406	*455*
Immunoglobulin G (g/L)	6.6–15.3	*21.5*	*24.4*	N/A	*21.9*	*19.9*	N/A	N/A	*23.2*
C-reactive protein (mg/L)	0–5	0.4	N/A	<0.2	<0.2	<0.2	<0.2	N/A	<0.2
ESR (mm/h)	0–10	*22*	N/A	*15*	8	*11*	9	9	*20*
Serum amyloid A (mg/L)	0.0–6.4	N/A	N/A	<4	N/A	N/A	N/A	N/A	N/A

N/A, not assessed; italic denotes above normal range, and bold denotes below normal range. *CD19^+^/CD27^+^/IgM^−^/IgD^−^; **CD3^−^/CD16^+^/CD56^+^.

Clinical trio exome sequencing revealed a de novo, heterozygous, likely pathogenic, N-terminal variant in the *MN1* gene (c.1306c>t, p.G436*). Premature truncation of *MN1* has previously been associated with the gain-of-function MN1 C-terminal truncation syndrome (23). N-terminal truncating variants are less reported and are expected to lead to nonsense-mediated decay resulting in loss-of-function and haploinsufficiency associated with mild learning problems, conductive hearing loss, growth delay, and dysmorphism, although individuals reported to date remain limited (24, 25, 26). While *MN1* haploinsufficiency likely explains our patient’s growth restriction and facial dysmorphism, this diagnosis is not consistent with the observed erosion of extremities, small joint arthritis, or immune abnormalities, such as elevated ESR and immunoglobulins (Table 2). Given these unexplained autoinflammatory features, the patient was referred to the Australian Autoinflammatory Diseases Registry for research investigation.

**Table 2.** Various aspects of the proband’s clinical presentation are consistent with previously reported *SAMHD1* deficiency or *MN1* haploinsufficiency cases

Proband genotype	*MN1* c.1306c>t, p.G436*		t(17;20)(p11.2;q11.23)	
Proband phenotype(s)	*MN1* haploinsufficiency	Reference	*SAMHD1* deficiency	Reference
**Developmental/sensory**				
Growth restriction	Consistent	(26)	Consistent	(27, 28)
Microcephaly	Consistent	(24)	Consistent	(27, 29, 30)
Facial dysmorphism	Consistent	(24, 26)	One case	(31)
Hoarse voice	Not reported	​	Consistent	(28, 32, 33)
Sensorineural hearing loss	Not reported	​	Not reported	​
Unilateral myopia	Not reported	​	Not reported	​
Late eruption of permanent teeth	Not reported	​	Not reported	​
**Cutaneous**				
Perniosis, acral ischemia	Not reported	​	Consistent	(22, 27, 28, 30, 34, 35, 36)
Dry or scaly skin (e.g., eczema)	Not reported	​	Consistent	(29, 34, 35)
**Immune/inflammatory**				
Elevated IFN signature	Not reported	​	Consistent	(20, 21, 22, 27, 30)
Small joint arthritis	Not reported	​	Consistent	(27, 28, 29, 30)
Mild anemia	Not reported	​	Consistent	(37)
Accelerated ESR	Not reported	​	Consistent	(27, 28)
Neutropenia	Not reported	​	Consistent	(37)
Hypergammaglobulinemia	Not reported	​	Consistent	(28, 34, 38)
NK cell lymphopenia	Not reported	​	Not reported	​
**Cardiovascular**				
Mitral regurgitation	Not reported	​	One case	(39)

**Figure 2.**
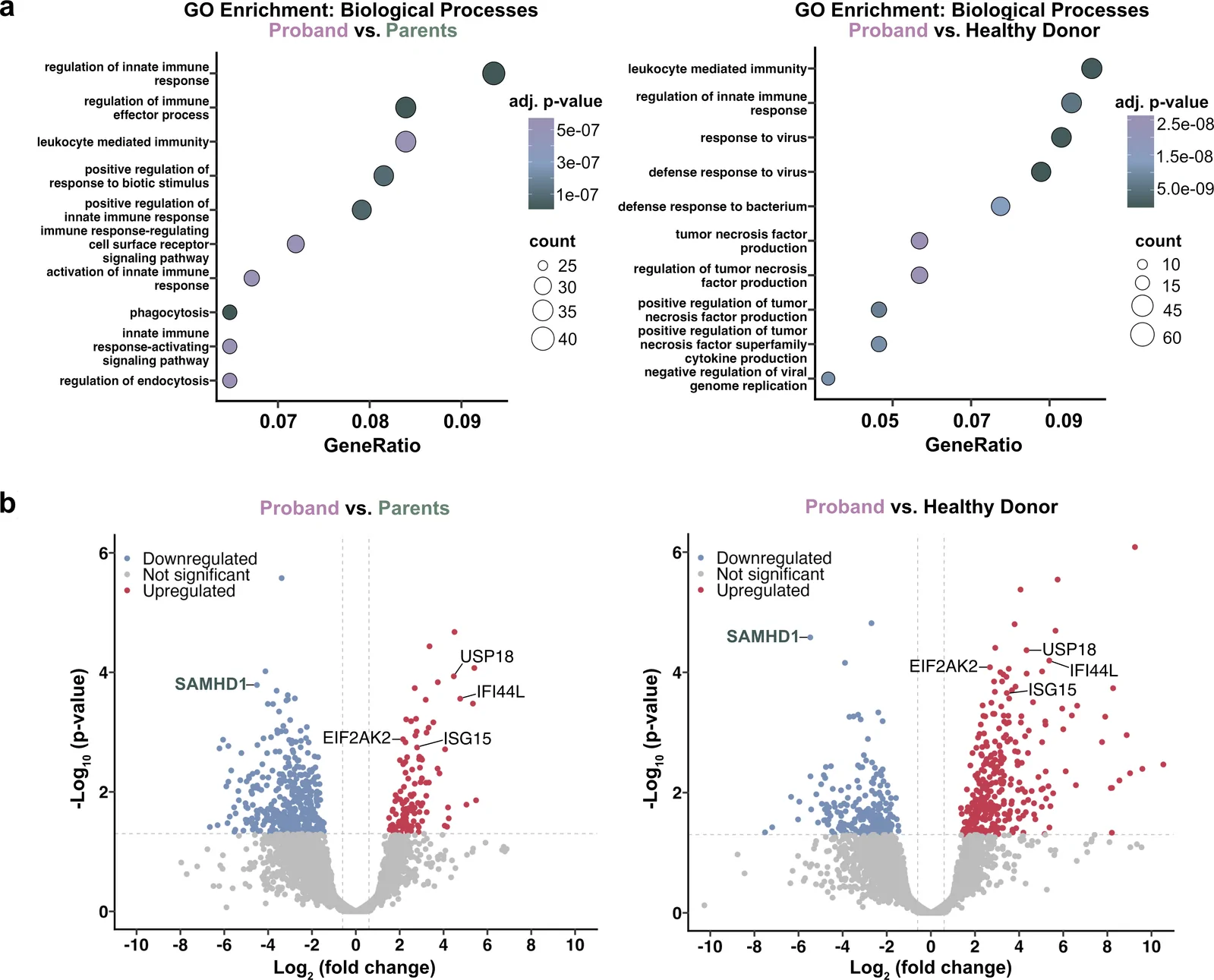
**Elevated innate immune transcriptional signature and reduced expression of *SAMHD1.*** RNA was extracted from PBMCs isolated from the patient, parents, or healthy donors; RNA-seq was performed, and DEGs were determined between the patient and parents (left panels) or the patient and healthy donors (right panels). **(a)** GO analysis of significant DEGs showing the 10 most differentially regulated pathways for each comparison. Overrepresented sets of DEGs are shown by the circle size and colored by the degree of statistical significance. **(b)** Volcano plots of candidate DEGs providing a magnitude measure of expression change (log_2_FC) and statistical significance (P value). P value <0.05 and absolute log_2_FC >0.6 are colored blue (downregulated) or red (upregulated). *SAMHD1* and a selection of common ISGs are labeled.

Bulk RNA sequencing (RNA-seq) was performed from the patient’s peripheral blood mononuclear cells (PBMCs) and compared to averaged parental samples. Gene Ontology (GO) enrichment analysis on the significant differentially expressed genes (DEGs) revealed altered expression of pathways including “regulation of innate immune response,” “regulation of immune effector process,” and “leukocyte-mediated immunity,” which are consistent with elevated innate immune activation and IFN-I responses. Similar analysis of the proband’s transcripts compared to those of healthy donor controls returned a nearly identical pattern with the addition of GO terms associated with responses to infectious agents such as viruses and bacteria, again suggesting immune activation and an IFN-I response (Fig. 2 a). Several ISGs were among the most upregulated significant DEGs when comparing the proband’s sample to the parental or healthy donor transcripts, including *IFI44L*, *ISG15*, *EIF2AK2*, and *USP18*; however, another known ISG, *SAMHD1*, was instead identified as one of the most significantly downregulated DEGs with P values of 1.6 × 10^−4^ and 2.3 × 10^−5^ when compared to parents and healthy donor datasets, respectively (Fig. 2 b). Following ISG filtering, we found that *SAMHD1* was the most significantly downregulated ISG (by >50-fold) in the patient vs. parents dataset and the only downregulated ISG when comparing the patient to healthy donor controls.

**Figure 3.**
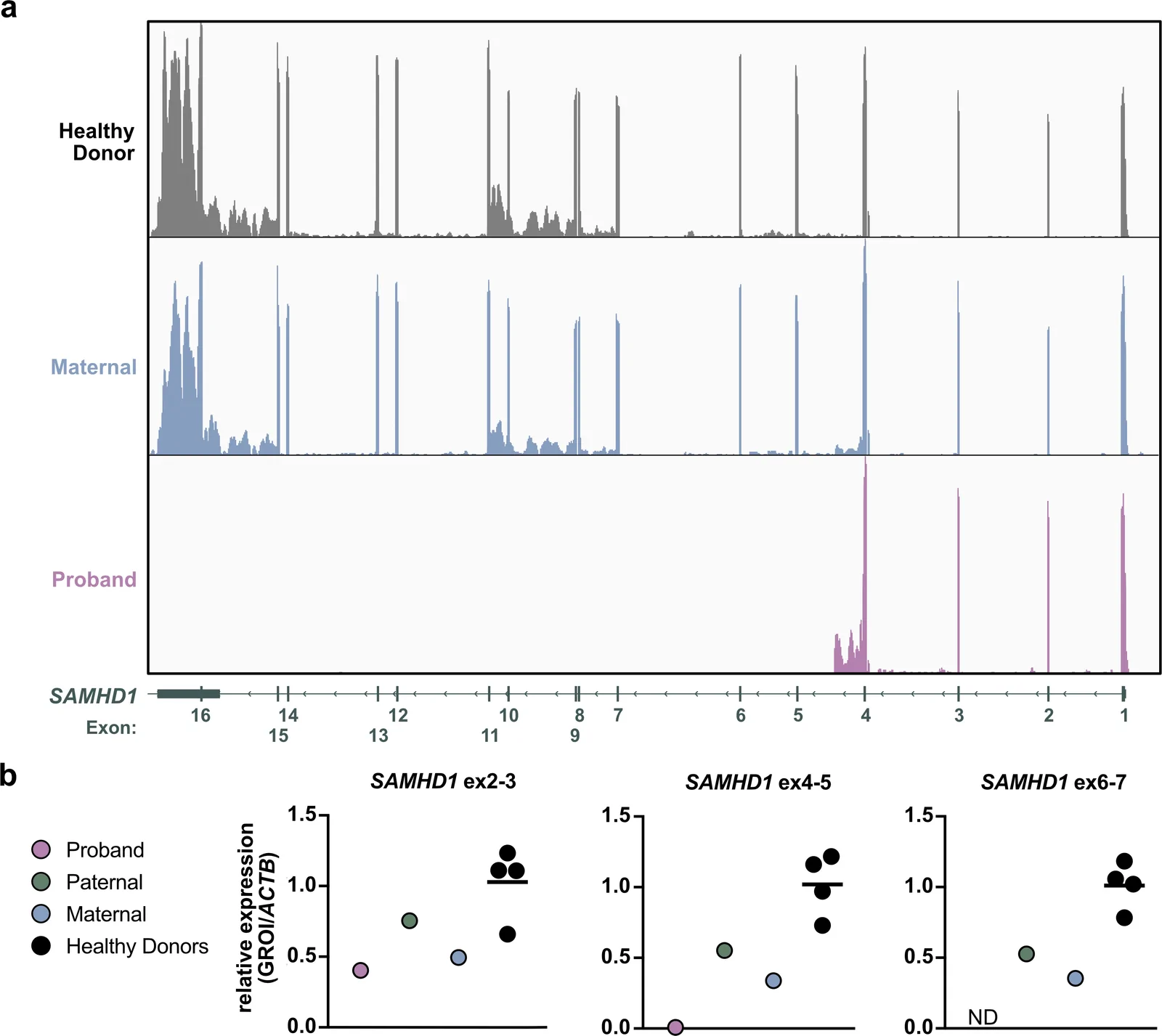
**3′ exons of *SAMHD1* are not transcribed in cells from the proband. (a)** Graphical representation of reads within the *SAMHD1* locus from RNA-seq performed on PBMCs from healthy donors, the mother, or the proband, showing a complete loss of reads 3′ of exon 4 in the proband (bottom track). **(b)** Relative expression of *SAMHD1* exons 2–3, exons 4–5, or exons 6–7 compared to *ACTB* as measured by qPCR using exon-specific primers on RNA isolated from PBMCs from the patient, both parents, or healthy donor controls (GROI, gene region of interest; ND, not detected).

Alignment of the RNA-seq reads within the *SAMHD1* locus revealed only a subset of reads to be downregulated in the patient, with reads through to exon 4, but not throughout the 12 exons 3′ of that locus. This was in contrast to complete reads throughout *SAMHD1* from the maternal sample (Fig. 3 a). This observation was confirmed by direct quantitative PCR (qPCR) using patient, parental, and healthy donor PBMC RNA and primers specific to exons 2–3, exons 4–5, or exons 6–7 of *SAMHD1*. This showed reduced expression of all the tested exons in both parental samples compared to healthy donors but further reduction of exons 4–5 and complete absence of exons 6–7 in the patient’s sample (Fig. 3 b), indicating a homozygous deletion of exons 4–7 and a heterozygous deletion in the parents.

Detection of early, but not late, exons of the *SAMHD1* transcript suggested that transcription of the 3′ end of the gene may have become uncoupled from the promoter in cells of the proband. Whole-genome sequencing was performed to investigate a suspected intronic variant following *SAMHD1* exon 4. Short-read sequencing revealed a suspected homozygous translocation, as zero reads from either direction mapped across the region. Investigation of soft-clipped bases showed mapping to poorly defined decoy sequences within the reference build. This indicated the other side of the potential translocation was possibly in a difficult-to-map region, such as repetitive sequence.

**Figure 4.**
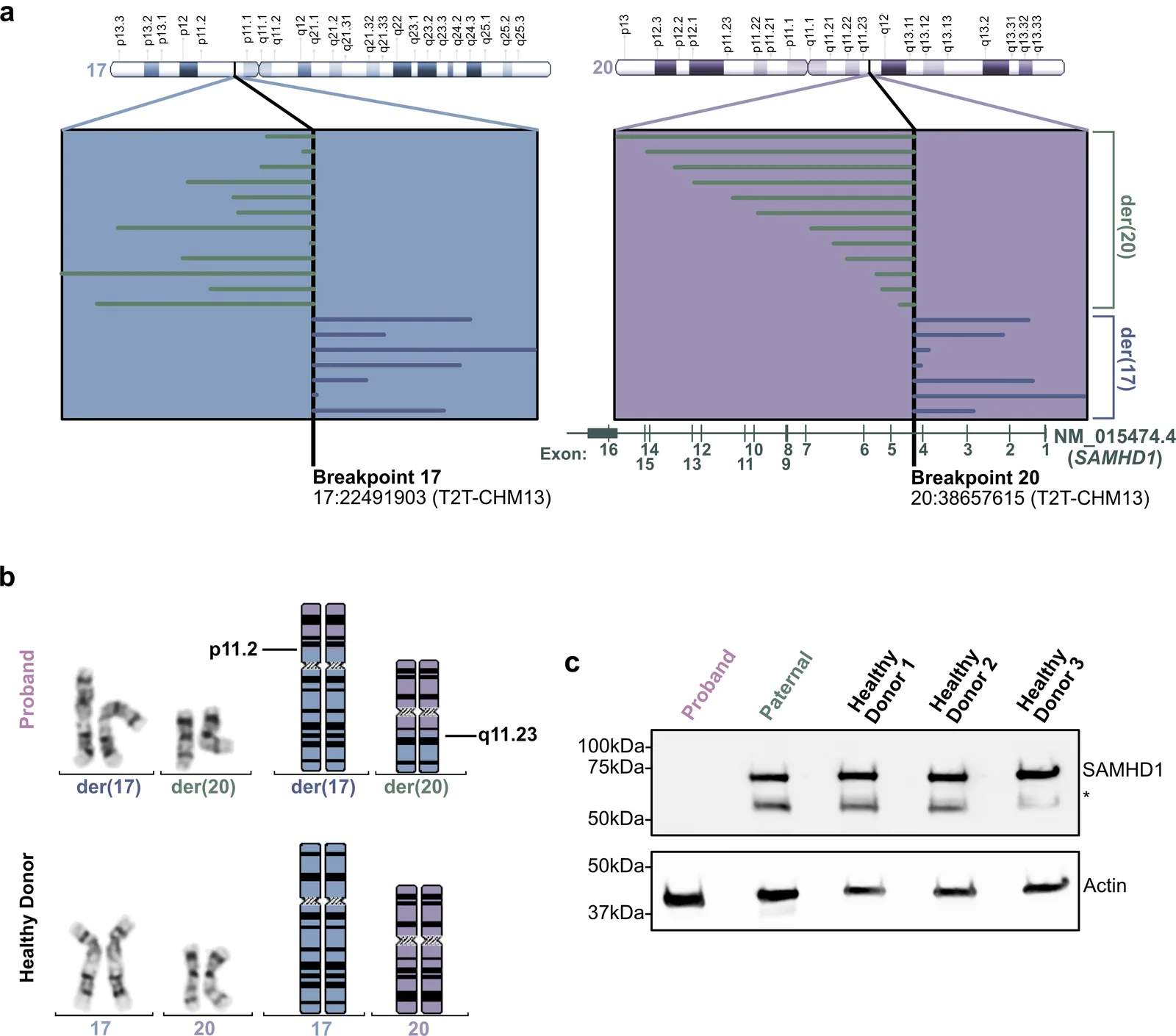
**A homozygous translocation between Chr17 and Chr20 results in loss of SAMHD1 expression in the proband. (a)** Graphical representation of long-read sequencing reads generated from PBMCs from the proband that cover the breakpoint loci on chromosomes 17 (blue) and 20 (violet). Reads from derivative 17 (der (17), navy lines) and derivative 20 (der (20), green lines) are labeled, as are the loci of the breakpoints (vertical black lines) and alignment to the *SAMHD1* gene. **(b)** Partial G-banding karyotype and corresponding ideograms of chromosomes 17 (blue) and 20 (violet) or their chromosomal derivatives (der) resulting from the translocation from peripheral blood T cells isolated from the proband or healthy controls. **(c)** Western blot of SAMHD1 and β-actin protein expression in PBMCs from the proband, the father, and healthy donors. (representative of 3 independent blots, * = lower molecular weight isoform of SAMHD1). Source data are available for this figure: SourceData F4.

Long-read genomic sequencing was performed; however, initial attempts to align the distal end of *SAMHD1* to the GRC38/hg38 reference genome were unsuccessful. Subsequent mapping to the telomere-to-telomere (T2T) reference genome (T2T-CHM13v2.0) (40) provided sufficient resolution within repetitive regions to reveal that the reciprocal breakpoint mapped proximal to the centromere in the p-arm of chromosome 17 (p11.2), a poorly annotated region in earlier human genome builds. Therefore, the proband was found to be homozygous for a balanced t(17;20)(p11.2;q11.23) translocation, with the chromosome 20q11.23 breakpoint occurring within intron 4 of the *SAMHD1* gene (Fig. 4 a). Chromosomal analysis was performed on a peripheral blood sample taken from the proband, and G-banding revealed a male karyotype with two identical, apparently balanced reciprocal translocations involving both copies of chromosomes 17 and 20, with breakpoints at 17p11.2 and 20q11.23, consistent with the long-read sequencing data (Fig. 4 b). To validate that this translocation event resulted in loss of SAMHD1 expression, western immunoblotting was performed on whole-cell lysate samples prepared from proband, paternal, and healthy donor PBMCs. While bands were detected at ∼72 kDa and 60–70 kDa in the paternal and healthy donor samples, corresponding to full-length SAMHD1 (UniProt entry Q9Y3Z3-1) and shorter isoforms (possibly UniProt entries Q9Y3Z3-3 or Q9Y3Z3-4), no bands were detected from the proband sample, suggesting complete loss of SAMHD1 protein expression (Fig. 4 c).

**Figure 5.**
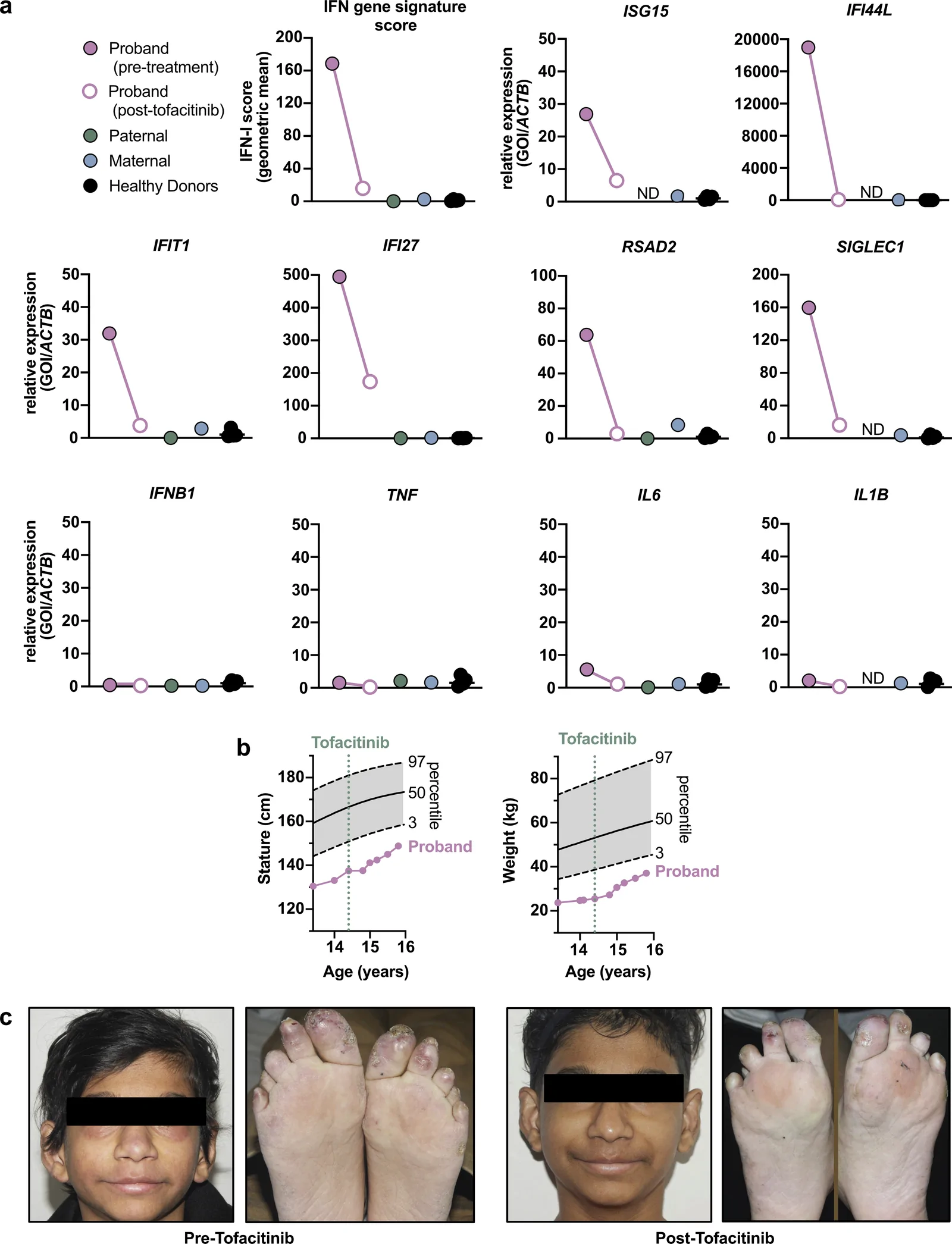
**Tofacitinib reduced transcriptional IFN signature and led to symptom improvement. (a)** 6-gene transcriptional IFN-I score performed on RNA from PBMCs taken from the indicated individuals (geometric mean). Genes included in the signature were measured by qPCR, and expression is plotted relative to *ACTB* for *ISG15*, *IFI44L*, *IFIT1*, *IFI27*, *RSAD2*, and *SIGLEC1*. Measurement of *IFNB1* and other non-IFN cytokine transcripts (*TNF*, *IL6*, and *IL1B*) was also performed (mean, pink line represents change in transcription from separate PBMC RNA samples taken from the proband before and after tofacitinib therapy; GOI, gene of interest; ND, not detected). **(b)** Growth charts tracking stature (left) or weight (right) of the proband (pink line) from 13.4 to 15.8 years old. Commencement of tofacitinib (green line) is indicated. Percentiles from the Centre for Disease Control are depicted by the grey shaded area. **(c)** Photographs of the proband before (left) and after (right) tofacitinib treatment. Lesions under the eyes and perniosis of the toes have improved with treatment.

The patient was treated with tofacitinib, a JAK inhibitor preferentially inhibiting JAK1 and JAK3, at doses extrapolated from other childhood inflammatory syndromes (41). qPCR analysis of a standardized subset of ISGs including *IFI27*, *IFI44L*, *IFIT1*, *ISG15*, *SIGLEC1*, and *RSAD2* confirmed the interferonopathy from which the patient suffered pre-treatment (Fig. 5 a). Both parental samples showed similar expression of ISGs to healthy donor controls. No increase was detected in *IFNB1* itself in the patient, which may reflect the transient and tightly regulated nature of IFN cytokine expression or elevation of IFN-I family members other than IFNβ. Little to no elevation of transcripts from other inflammatory pathways was observed, including *TNF*,* IL6*, and *IL1B*. Similar samples taken from the patient following tofacitinib treatment illustrated the efficacy of JAK inhibition in suppression of this IFN signature (Fig. 5 a). This correlated with marked improvement of symptoms following tofacitinib treatment, including improved weight and accelerated growth trajectory (Fig. 5 b), decreased perniosis (Fig. 5 c), and improved joint range of motion with a complete resolution of active joint count from 14 pre-tofacitinib to 0 after treatment. NK cell numbers were completely normalized, and switched memory B cells increased by more than threefold (Table 1). Treatment with a more targeted therapy, anifrolumab, an IFN α receptor 1 antagonist, was considered but declined owing to the degree of improvement while on tofacitinib.

## Discussion

Exon-specific transcriptional analysis indicated complete loss of expression of the 3′ end of *SAMHD1* in the proband. Due to the loss of the *SAMHD1* 3′ untranslated region, stability of the truncated transcript is expected to be severely reduced, and western blotting showed complete loss of protein product in patient PBMCs. While it is possible that truncated SAMHD1 protein does not contain the epitope detected by the antibody used in this study, loss of *SAMHD1* expression beyond exon 4 is predicted to result in ablation of the HD domain, implying any undetected protein product would be deficient in dNTP triphosphatase activity. Similar analysis of the parental samples showed only a partial loss of expression of the 3′ end of the transcript, suggesting that both parents likely possess single copies of the chromosomal derivatives arising from the t(17;20)(p11.2;q11.23) translocation and subsequently are heterozygous carriers of the disrupted *SAMHD1* allele. Further interrogation of the parental samples, however, showed no defect in SAMHD1 protein expression, nor regulation of ISGs, implying that the autoinflammatory phenotype in the proband is recessive.

Other cases of SAMHD1 deficiency have been reported with a broad spectrum of symptoms (29). At a single center, three cases with identical homozygous *SAMHD1* c.625G>A, p.G209S variants, presented with a highly divergent clinical spectrum, including both FCL and AGS (42). Therefore, it is likely that there are environmental or genetic phenotypic modifiers of the symptomatic spectrum observed in SAMHD1-deficient individuals. Among interferonopathies, there is precedent for phenotypic modifier alleles, such as the HAQ allele of *STING1,* one of a number of factors that may suppress the clinical presentation of COPA syndrome (43). Further interrogation of whole genome sequencing from our patient shows that he is also heterozygous for the *STING1* HAQ allele, so the lack of typical AGS5 symptoms (e.g., encephalopathy, intellectual developmental delay) despite homozygous loss of *SAMHD1* expression may be partially attributable to a similar suppressive effect of this haplotype of *STING1*. However, in the absence of a larger cohort of SAMHD1-deficiency patients to confirm such a correlation, this remains hypothetical. Furthermore, analysis of a broader cohort of COPA syndrome patients has uncovered individuals without the *STING1* HAQ allele who are asymptomatic and one HAQ allele carrier who suffers from *COPA*-driven kidney disease, indicating that the HAQ allele is not the only factor modulating penetrance or severity of STING-related interferonopathies (44). Indeed, the clinical presentation of the current proband may be influenced by a number of other genetic or environmental factors (45), especially considering that he also carries a de novo variant in *MN1*. Of the symptoms reported from the proband in this study, most are consistent with previously reported cases of recessive loss-of-function variants in *SAMHD1*, with only growth restriction, microcephaly, and mild facial dysmorphism being consistent with previous reports of *MN1* haploinsufficiency (Table 2).

Balanced reciprocal translocations occur in 0.09% of unselected newborns (46). Each individual translocation within this group is exceedingly rare, with most individual non-Robertsonian translocations being represented at most once with any given cohort (47). The autozygous balanced nature of the current proband’s genotype is also remarkable, considering the probability of two parents carrying an identical balanced t(17;20)(p11.2;q11.23) translocation is incredibly low, making the genomic architecture of the proband strikingly unique. In this family, there is potential fourth-degree consanguinity, meaning that the balanced translocation has been successfully transmitted at least three times on each side. It remains technically possible that the balanced translocation could be present in the ancestral population at a low frequency; however, the long regions of homozygosity and potential consanguinity within this family mean the translocated allele is much more likely to have arisen from a recent shared ancestor.

We are unaware of any heterozygous germline t(17;20)(p11.2;q11.23) translocations in the literature. One other report of a homozygous t(17;20) translocation has been published; however, as the breakpoints were between distinct areas of both affected chromosomes (t(17;20)(q21.1;p11.21)), the severe developmental abnormalities in that homozygous neonate are unrelated to those in the proband in this study (48). The most recent meta-analysis of homozygosity in balanced reciprocal translocations was published in 2010, with only five families recorded at the time and very few additional case reports since (49). The likelihood that reports of homozygosity only arise upon investigation of pathology, as was the case for the family reported here, makes determining the true rate of homozygous reciprocal translocation, including those who are phenotypically normal, a challenging exercise.

**Figure S1.**
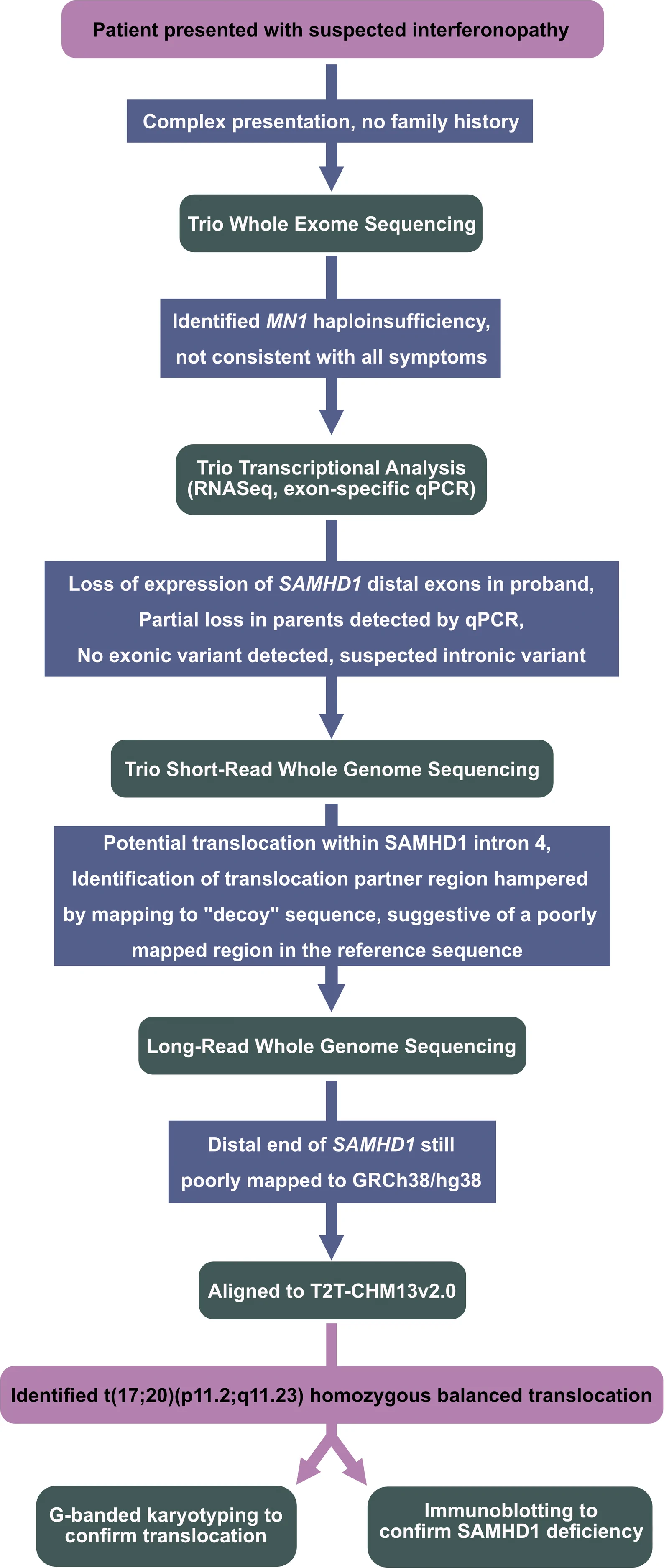
**Flow chart showing iterative genetic testing of the proband.** Iterative clinical and laboratory-based genetic testing was used to identify an uncommon homozygous balanced translocation in the proband from this study. An iterative approach such as that applied here can assist with identifying rare and unexpected variants from patients with inborn errors of immunity; however, this exact regimen is provided only as an example. The specific genetic tests and clinical decisions required should be assessed on a case-by-case basis in consultation with a multidisciplinary care team.

This appears to be only the third pathogenic autosomal recessive gene disruption event due to a homozygous balanced translocation described (50, 51). Identification of this balanced translocation causing an inborn error of immunity highlights the utility in certain cases of iterative genetic testing, including transcriptome analysis and long-read sequencing (Fig. S1). While the approach we have described was valuable for identification of the structural variation carried by this patient, the particular suite of genetic tests and decisions required to identify other rare inborn errors should be assessed on a case-by-case basis. Nonetheless, because immune-related genes are often rapidly evolving and typically nonessential for survival, it is plausible that, in some cases, disease may be driven by exceptionally rare or otherwise improbable genetic alterations that require nonstandard genomic diagnostics such as those employed here.

## Materials and methods

### Patient cells

PBMCs were isolated from the patient and family members at Monash Health and from healthy donors at the Walter and Eliza Hall Institute or Red Cross Lifeblood. PBMCs were isolated from whole blood using Ficoll (GE Healthcare) before being frozen in fetal bovine serum (Bovogen) with 10% DMSO (Sigma-Aldrich) and either used directly or stored in liquid nitrogen until use (cell handling between groups was consistent within each experiment).

### Whole exome sequencing

Buccal swab samples were taken from the proband and both parents, and exome sequencing was performed by Blueprint Genetics Whole Exome Family Plus Test (v3, 2023) utilizing paired-end 150 bp reads on the Illumina platform. Reads were mapped to the *Homo sapiens* genome (GRCh37/hg19) using Burrows-Wheeler Aligner (BWA-MEM) software (52).

### Real-time qPCR

1 × 10^7^–4 × 10^7^ PBMCs were lysed using TRIzol reagent (Invitrogen) for 5 min at room temperature. Samples were either stored at −80°C or immediately processed. RNA extraction was performed as per the manufacturer’s instructions. cDNA was generated from 1 to 2 μg of total RNA using the Invitrogen SuperScript III First-Strand Synthesis System for reverse transcription PCR (Invitrogen) utilizing oligo(dT)s. qPCR was performed using SYBR Green/ROX qPCR Master Mix (Thermo Fisher Scientific) on ViiA 7 or QuantStudio 6 Real-Time PCR systems (Thermo Fisher Scientific). Primers for genes of interest and housekeeping genes are listed in Table 3. Each sample was run in duplicate, and samples were normalized using the housekeeping gene *ACTB*. Results were analyzed using the ΔΔCt method and represented as fold change (FC) of the average of healthy donor samples from the same experiment.

**Table 3.** qPCR primer sequences

Target	Forward Sequence	Reverse sequence
h*IFI27*	5′-GCC​ACA​ACT​CCT​CCA​ATC​AC-3′	5′-ATC​AGC​AGT​GAC​CAG​TGT​GG-3′
h*IFI44L*	5′-ACT​AAA​GTG​GAT​GAT​TGC​AG-3′	5′-TGC​AGA​GAG​GAT​GAG​AAT​ATC-3′
h*IFIT1*	5′-ATC​CAC​AAG​ACA​GAA​TAG​CCA-3′	5′-CCA​GAC​TAT​CCT​TGA​CCT​GAT-3′
h*ISG15*	5′-ACA​GCC​ATG​GGC​TGG​GA-3′	5′-CTT​CTG​GGT​GAT​CTG​CGC​CT-3′
h*RSAD2*	5′-CTC​TGT​GGA​GGA​GCC​TGG​TC-3′	5′-AGT​TGA​TCT​TCT​CCA​TAC​CAG​CTT-3′
h*SIGLEC1*	5′-TGC​TCC​GTG​TGC​TCT​ACC​CT-3′	5′-CAC​CGA​ATT​CTC​GCC​CAT​GC-3′
h*IFNB1*	5′-TGT​CGC​CTA​CTA​CCT​GTT​GTG​C-3′	5′-AAC​TGC​AAC​CTT​TCG​AAG​CC-3′
h*TNF*	5′-TCT​CTC​AGC​TCC​ACG​CCA​TT-3′	5′-CCC​AGG​CAG​TCA​GAT​CAT​CTT​C-3′
h*IL6*	5′-TCA​ATA​TTA​GAG​TCT​CAA​CCC​CCA-3′	5′-GAA​GGC​GCT​TGT​GGA​GAA​GG-3′
h*IL1B*	5′-ATG​ATG​GCT​TAT​TAC​AGT​GGA​AA-3′	5′-GTC​GGA​GAT​TCG​TAG​CTG​GA-3′
h*ACTB*	5′-GCG​AGA​AGA​TGA​CCC​AGA​TC-3′	5′-CCA​GTG​GTA​CGG​CCA​GAG​G-3′
h*SAMHD1* exon 2–3	5′-TCA​CAG​GCG​CAT​TAC​TGC​C-3′	5′-GGA​TTT​GAA​CCA​ATC​GCT​GGA-3′
h*SAMHD1* exon 4–5	5′-CCT​CTC​CTC​GTC​CGA​ATC​ATT-3′	5′-CCA​CCC​CTA​GAC​TAT​GCT​CAA-3′
h*SAMHD1* exon 6–7	5′-GTC​ATG​GGC​CAT​TTT​CTC​AC-3′	5′-CTG​AGC​CTT​GTT​CAT​GCG​TCC-3′

### ISG score calculation

ISG Score was calculated from SYBR green qPCR results for the following ISGs: *IFI27*, *IFI44L*, *SIGLEC1*, *ISG15*, *IFIT1*, and *RSAD2* (20, 53). Expression of each ISG was calculated using the formula 2^−ΔΔCt^, then normalized to the average of *ACTB* expression. For each ISG, individual samples were then normalized to the average of all healthy donors run in the experiment. Finally, the geometric mean of the relative expression of these six ISGs was used to calculate the final IFN score.

### RNA-seq and transcriptional analysis

RNA was extracted from PBMCs isolated from the patient, parents, and healthy donors, as described above, and sent for sequencing by the Australian Genome Research Facility. mRNA was purified using oligo(dT) beads and, following fragmentation, the cDNA library was prepared using the Illumina TruSeq stranded RNA protocol. Sequencing using the Illumina NovaSeq X Plus platform was performed to generate paired-end 150 bp reads. Base calling was performed with Illumina DRAGEN BCL Convert (07.021.645.4.0.3 pipeline). Following demultiplexing and quality control, sequences were mapped to the *H. sapiens* genome (GRCh38/hg38) using the STAR aligner (v2.3.5a) (54). A gene counts matrix was assembled using the featureCounts v1.5.3 utility of the Subread package (55). StringTie (v2.1.4) was employed to obtain estimated transcript structure and abundance from aligned BAM files (56). Differential expression analysis was performed using a generalized linear model in the edgeR package (v4.0.9) (57) using R (v4.3.1) (58). GO analysis was completed with the org.Hs.eg.db package (59) to compare the significant genes (P value <0.05) to the genome-wide annotation for the Human database; clustering genes based on Biological Processes (Ont. = BP). Overrepresented sets of DEGs are shown by the circle size and colored by the degree of statistical significance. Differential gene expression analysis compared a predetermined significance threshold and expression FC. To capture all potential genes of interest, a standard P value <0.05 was used to highlight statistical significance, and absolute log_2_FC >0.6 was used to take a sensitive approach to identifying the FC expression of genes. Volcano plots were created using base R (v4.5.1) plotting functions (60), and those above the assigned significance threshold were colored in blue and red to represent downregulated and upregulated genes, respectively. ISGs were identified within the lists of significant DEGs by first filtering against the Interferome database (61) with the search conditions “type-I IFN,” “in vivo,” and “*H. sapiens.*” To further filter for genes directly induced by IFN signalling, the Interferome-filtered DEGs were then cross-checked against genes with STAT1-binding sites within 1 kb of the transcriptional start site, generated from the ChIP-Atlas Target Genes tool (62), selecting dataset SRX150550 (K562 cells treated for 6 h with IFNα).

### Whole genome sequencing

Short-read whole-genome sequencing was performed on the proband and both parents by the Garvan Genomics Platform, using an Illumina NovaSeq 6000 and Illumina DNA PCR-Free Prep Kit. Genomes were sequenced to a mean coverage of ≥30×, and paired-end reads were aligned to the human genome reference sequence (GRCh37).

### Long-read sequencing and analysis

Library preparation and Oxford Nanopore Technologies sequencing were performed by the Garvan Genomics Platform. DNA was first extracted from patient PBMCs using the PacBio PanDNA kit. High molecular weight genomic DNA was then sheared to ∼20–30 kb fragment size using the Diagenode Megaruptor 3 DNA shearing system (speed 29) and visualized on an Agilent Femto Pulse using the Genomic DNA 165 kb Kit prior to library preparation. The sequencing library was generated using the SQK-LSK114 ligation sequencing kit with 3 µg of sheared DNA as input. Sequencing was performed on a PromethION run with a FLO-PRO114M flow cell with MinKNOW (v25.03.7, MinKNOW Core v6.4.8). High-accuracy basecalling was performed using Dorado (v7.8.3, basecalling module version dna_r10.4.1_e8.2_400bps_hac@v4.3.0) and Bream (v8.4.4) to generate raw reads in FASTQ format. Quality filtering was performed to retain only those reads with a minimum Q score of 9.

Raw reads were quality trimmed using filtlong (v0.3.1, Python v3.10.14) (63) with parameters --min_length 1000, --keep_percent 90, and --target_bases 40000000000 to preserve approximately the best-quality half of all bases. Alignment of reads to the human T2T reference genome (T2T-CHM13v2.0) (40), and structural variant calling were performed using NanoVar (v1.8.3, with Python v3.11.13) (64) in Oxford Nanopore mode (-x ont, otherwise default parameters), which calls Minimap2 (v2.30-r1287) (65) for alignment steps. The aligned reads file was sorted using samtools (v1.22) sort, and an index file was generated using samtools index with default parameters for viewing within Interactive Genomics Viewer (66).

### Karyotype studies

G-banded karyotype analysis was performed on phytohemagglutinin-stimulated T cells from this patient using standard methods.

### Molecular karyotyping

DNA was extracted from a peripheral blood sample using the EZ1 DSP Blood kit (QIAGEN), and microarray was performed using the Illumina Infinium GSA-24 v3.0. Analysis was performed on NxClinical (Bionano) using data normalized by Genome Studio (Illumina).

### Immunoblotting

PBMCs were isolated using Ficoll (GE Healthcare) as described above and underwent RBC lysis in RBC lysis buffer (156 mM ammonium chloride, 11.9 mM sodium bicarbonate, and 0.1 mM EDTA) for 5 min at room temperature before dilution to 30 ml with 1× PBS, followed by washing and resuspension in PBS for counting. 2 × 10^6^ cells were lysed in 1× radioimmunoprecipitation assay buffer (1% Triton X-100, 20 mM tris-HCl [pH 7.4], 150 mM sodium chloride, 1 mM EDTA, 0.5% sodium deoxycholate, 3.5 mM SDS, and 10% glycerol) supplemented with 10 mM sodium pyrophosphate, 5 mM sodium fluoride, 1 mM sodium orthovanadate, 1 mM phenylmethylsulfonyl fluoride and cOmplete protease inhibitors (Roche Biochemicals) for 30 min at 4°C. Samples were processed through Pierce centrifuge columns (Thermo Fisher Scientific) to shred genomic DNA. After addition of reducing SDS sample loading buffer (1.25% SDS, 12.5% glycerol, 62.5 mM tris-HCl [pH 6.8], 0.005% bromophenol blue, and 50 mM dithiothreitol) and denaturation at 95°C for 5–10 min, samples were separated on Bolt 4–12% precast SDS-PAGE gels (Thermo Fisher Scientific) with MES running buffer (Thermo Fisher Scientific) and subsequently transferred onto nitrocellulose membrane (Millipore). Membranes were blocked in 5% skim milk in Tris-buffered saline (TBS) containing 0.1% Tween 20 (Sigma-Aldrich) (TBS-T) before overnight incubation with specific primary antibody in 5% skim milk in TBS-T at 4°C: Rabbit anti-SAMHD1 (1:1,000 #12586-1-AP; Proteintech). Membranes were washed five times with TBS-T and incubated with appropriate HRP-conjugated secondary antibodies: Goat anti-Rabbit (1:10,000, #P044801-2; Agilent) or anti-actin–HRP (1:10,000, #sc-47778; Santa Cruz Biotechnology) and washed five times again. Finally, membranes were developed using Immobilon Forte Chemiluminescent HRP Substrate (Millipore) and imaged using the ChemiDoc Touch Imaging System (Bio-Rad).

### Online supplemental material

Fig. S1 shows the iterative testing workflow and reasoning behind each step used to identify the uncommon structural genetic variant carried by the proband in this study.

## Ethics statement

Informed consent was obtained from the patient and family, in accordance with local regulations, and a protocol for research on human subjects was approved by the institutional review boards of Monash Health (Human ethics number: HREC-15-MonH-31) and the Garvan Institute of Medical Research (Human ethics number: HREC X20-0177). Healthy donor samples were obtained from the Volunteer Blood Donor Registry at the Walter and Eliza Institute of Medical Research (Human ethics number: WEHI HREC 18/07) or the Australian Red Cross Lifeblood (Human ethics number: MonH-2025-483166). All participants, or their legal representatives, consented to take part in this study and to have the results of this research published, including patient images.

## Supplementary Material

SourceData F4 is the source file for Fig. 4.

## Data Availability

The raw genetic and transcriptional data underlying this study are not publicly available, given it is classified as protected health information. The data are available from the corresponding author upon reasonable request.
