# Supplementary material for: Iterative genetic testing identifies SAMHD1 deficiency caused by a homozygous balanced translocation
Source: J Hum Immun. 2026 Aug 26;2(6):e20260044. doi: 10.70962/jhi.20260044 (PMC13510289; doi:10.70962/jhi.20260044)

SOURCE DATA FOR FIGURE 4C

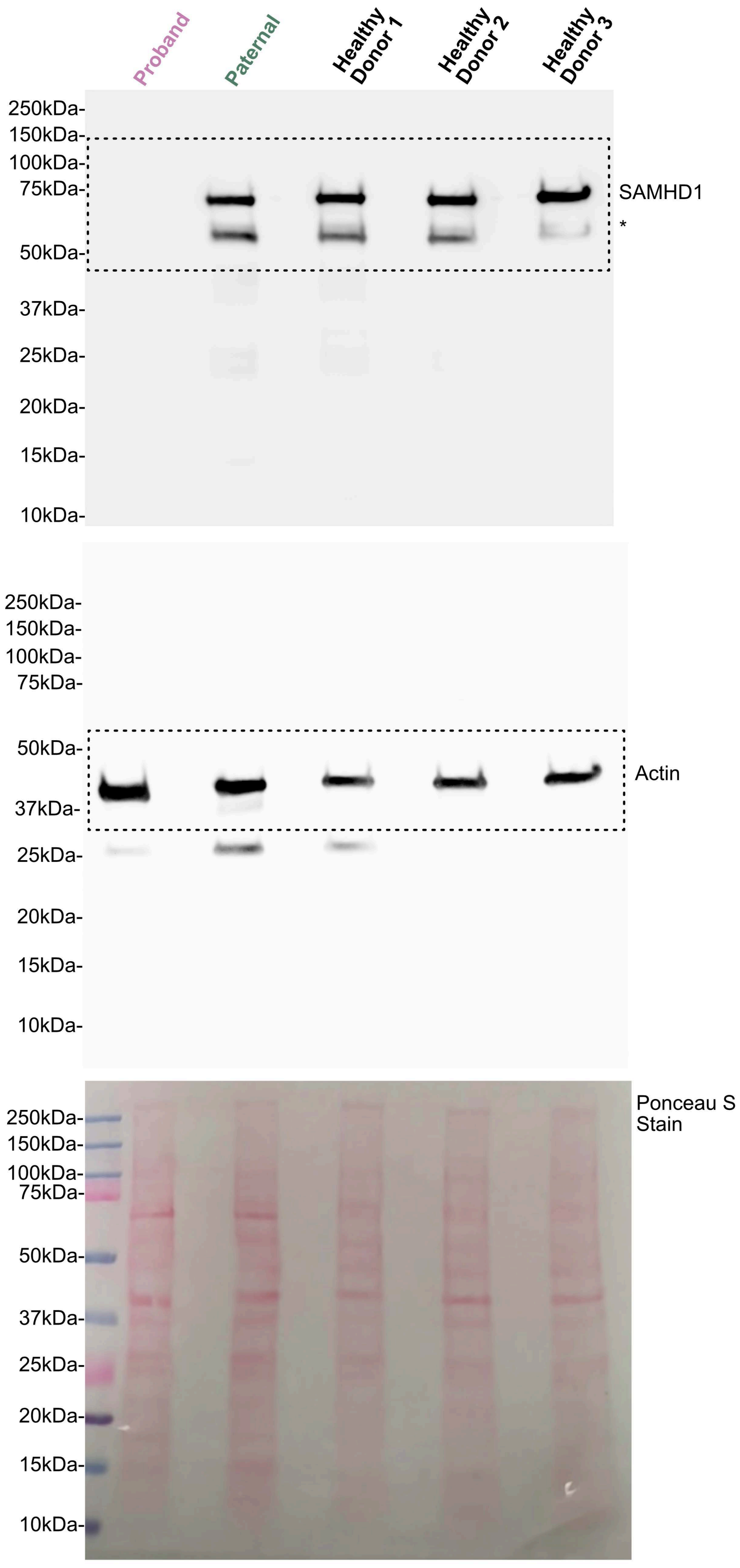

Supplement: Supplementary file 1 [file jhi_20260044_sourcedataf4.pdf]
